# Surgical and per-oral endoscopic myotomy (POEM) for the treatment of primary esophageal motility disorders: A systematic analysis of current trends in Germany between 2011 and 2019

**DOI:** 10.1371/journal.pone.0297265

**Published:** 2024-01-23

**Authors:** Jennis Kandler, Tobias Essing, David Schöler, Georg Flügen, Wolfram T. Knoefel, Christoph Roderburg, Tom Luedde, Sven H. Loosen

**Affiliations:** 1 Medical Faculty of Heinrich Heine University Düsseldorf, Department of Gastroenterology, Hepatology and Infectious Diseases, Düsseldorf, Germany; 2 Department of Internal Medicine II, Marien Hospital, Wesel, Germany; 3 Medical Faculty of Heinrich Heine University Düsseldorf, Department of General, Visceral and Pediatric Surgery, University Hospital Düsseldorf, Düsseldorf, Germany; Kyung Hee University School of Medicine, REPUBLIC OF KOREA

## Abstract

**Background/Aims:**

While surgery remains a standard treatment for primary esophageal motility disorders (PEMDs), per-oral endoscopic myotomy (POEM) has recently evolved as an alternative. Systematic data on current trends of invasive procedures for PEMDs in Germany are missing.

**Methods:**

Hospital discharge data were used to evaluate trends and mortality of invasive treatment options for PEMDs in Germany between 2011 and 2019.

**Results:**

4543 cases of PEMDs (achalasia: n = 4349, dyskinesia of the esophagus: n = 194) receiving open surgery (n = 200), minimal invasive surgery (n = 2366), or POEM (n = 1977) were identified. The relative proportion of POEM significantly increased from 10.9% (2011) to 65.7% (2019). Hospital mortality was 0.2%. The median duration of mechanical ventilation was significantly lower in POEM patients (29.4 hours) compared to open (274.0 hours) or minimal invasive (91.9 hours) surgery. The duration of hospitalization was lowest among POEM patients (5.7 days) compared to surgical procedures (13.7 and 7.7 days).

**Conclusion:**

While the low in-hospital mortality of all procedures combined confirms the solid safety profile of invasive procedures in general, our findings show that POEM has the lowest duration of mechanical ventilation and hospitalization compared to invasive surgical options.

## Introduction

Primary esophageal motility disorders (PEMDs), including idiopathic achalasia and spastic esophageal disorders (SEDs), are a group of diseases in which impaired swallowing occurs due to an alteration in esophageal neuromuscular structures. With an estimated annual incidence of 1 to 3 cases per 100,000 individuals, achalasia is considered a rare disease [1–3]. The etiology remains still unclear. Presumably, viral infections might trigger autoimmunological processes, causing chronic inflammation in the inhibitory neurons in the esophageal myenteric plexus, leading to selective loss of these neurons [4]. This causes cessation of the relaxation of the lower esophageal sphincter (LES) as well as the propulsive peristalsis of the tubular esophagus, resulting in an insufficient relaxation of the LES combined with a lack of esophageal peristalsis [5]. Main symptoms of achalasia include dysphagia, regurgitation, retrosternal pain, and weight loss, which have a considerable negative impact on the quality of life of patients [6]. SEDs such as esophageal spasm (DES), Jackhammer esophagus (JE), and esophagogastric junction out-flow obstruction (EGJOO) are a rare group of PEMDs that, given the introduction and evolution of high-resolution manometry (HRM), are now better characterized and lead to symptoms quite similar to those of achalasia [7–9].

PEMDs traditionally were treated pharmacologically (e.g., calcium channel blockers), endoscopically (pneumatic dilatation, PD; injection of Botulinum toxin A, BTX), or surgically (myotomy). In achalasia, a step-up approach had been established. In most cases, PD of the LES was used as the primary treatment, followed by a surgical myotomy in refractory cases [10]. PD is a minimally invasive procedure with a long-term therapeutic success rate between 50% and 85% [10]. However, a major disadvantage of PD is that repetitive dilations are required in up to 25% to achieve these success rates [11]. Surgical therapy for achalasia consists of a myotomy of the LES, which is nowadays performed laparoscopically (laparoscopic Heller myotomy, LHM) [12] providing good functional results but without superiority over PD [13,14]. In 2008, per-oral endoscopic myotomy (POEM) was introduced as an endoscopic, incisionless alternative to LHM for the treatment of achalasia [15]. POEM has shown excellent clinical response rates (80% to 90%), including long-term follow-up studies [10,13,16–19], with limited numbers of serious adverse events that usually can be treated intraprocedurally [20]. In achalasia, POEM is superior to PD and non-inferior to LHM [10,13,19,21,22]. Given the general lack of standardized treatment strategies as well as the limited efficacy of nonsurgical treatment options for SEDs [23,24], POEM has also recently been investigated with promising results for the treatment of SEDs [25–32].

However, systematic data on current trends of invasive treatment strategies for PEMDs in Germany are widely missing. In the present manuscript, we therefore used standardized hospital discharge data from the Federal Statistical Office of Germany to evaluate current clinical developments as well as the hospital mortality of invasive PEMD treatments between 2011 and 2019.

## Materials and methods

### Study design

This study aims at the retrospective evaluation of recent trends, hospital mortality and associated clinical parameters of invasive treatment procedures for esophageal motility disorders in Germany. The analyses are based on standardized hospital discharge from 2011 to 2019 that were provided by the Federal Statistical Office of Germany (Wiesbaden, Germany). To access the data via remote data extraction an official contract was signed between the University Hospital Düsseldorf/Medical Faculty of the Heinrich-Heine-University and the Federal Statistical Office in Wiesbaden in 2021. Ethical approval was granted by the Ethics Committee at the Medical Faculty of Heinrich Heine University Düsseldorf under the study number 2022–1856. The authors had no access to information that could identify individual participants during or after data collection.

### Patient eligibility criteria and variables

We identified and grouped patients undergoing invasive treatment procedures for esophageal motility disorders during the observation period using the OPS codes for a) open esophagomyotomy/ esophago-gastromyotomy (open heller myotomy [OHM], OPS 542000, 542001, 542004, 542005, 542020, 542021, 542024, 542025,) “minimal invasive” esophagomyotomy/ esophago-gastromyotomy (laparoscopic or thoracoscopic Heller myotomy [LHM], OPS 542002, 542003, 542022, 542023) and c) endoscopic esophagomyotomy/ esophago-gastromyotomy (per-oral endoscopic myotomy [POEM]; OPS 542006, 542026). Only patients with primary diagnosis of an esophageal motility disorder (K22.0: Achalasia of cardia or K22.4: Dyskinesia of oesophagus) were included. K22.4 applies to the diagnosis of “corkscrew esophagus”, “diffuse esophageal spasm” and “spasm of the esophagus”. Hospital mortality was evaluated by the coded discharge type "death" (entl_grd = 7) in relation to the other discharge types. Additionally, we analyzed the duration of hospital stay (days) and the mean duration of mechanical ventilation (hours). The mean duration of mechanical ventilation was only evaluated for patients who required prolonged mechanical ventilation after the procedure itself. Tables 1 and S1 provide detailed information on the study population.

**Table 1:** Characteristics of study population.

	Study population
**Total number of patients**	4,543
Hospital death [total]	10
Hospital mortality rate [%]	0.22%
**Sex** [total]	
male	2,494
female	2,049
**Age** [Mean and SD]	49.36 (17.95)
Age group [total]	
0–17 Years	161
18–30 years	649
31–50 years	1,551
51–70 years	1,504
>70 years	678
**Federal state** [total]	
Baden-Württemberg	327
Bavaria	886
Berlin	117
Brandenburg	13
Bremen	22
Hamburg	809
Hesse	351
Lower Saxony	145
Mecklenburg-Western Pomerania	17
North Rhine-Westphalia	1,354
Rhineland-Palatinate	194
Saarland	6
Saxony	218
Saxony-Anhalt	42
Schleswig-Holstein	12
Thuringia	30
**Disease** [total]	
Dyskinesia of esophagus	194
Achalasia of cardia	4,349
**Procedure** [total]	
Open heller myotomy (OHM)	200
Lap./thorac. Heller myotomy (LHM)	2366
per-oral endoscopic myotomy (POEM)	1977

### Statistical analysis

Analyses were performed via remote data access at the Federal Statistical Office of Germany (Wiesbaden, Germany) using SPSS (v23.0, IBM Corporation, Armonk, USA) and Excel (v16.71, Microsoft, Redmond, USA). Descriptive analyses are based on cross-tabulations. The comparison between binary variables (e.g., hospital death yes/no) were performed using Pearson’s chi-square test [33]. Variations of the dependent and independent variables over time were analyzed using Pearson’s R and linear regression. All statistical tests were two-sided. A two-sided p-value of p<0.05 was considered statistically significant.

## Results

### Current trends of invasive treatments for esophageal motility disorder in Germany between 2011 and 2019

**Fig 1:**
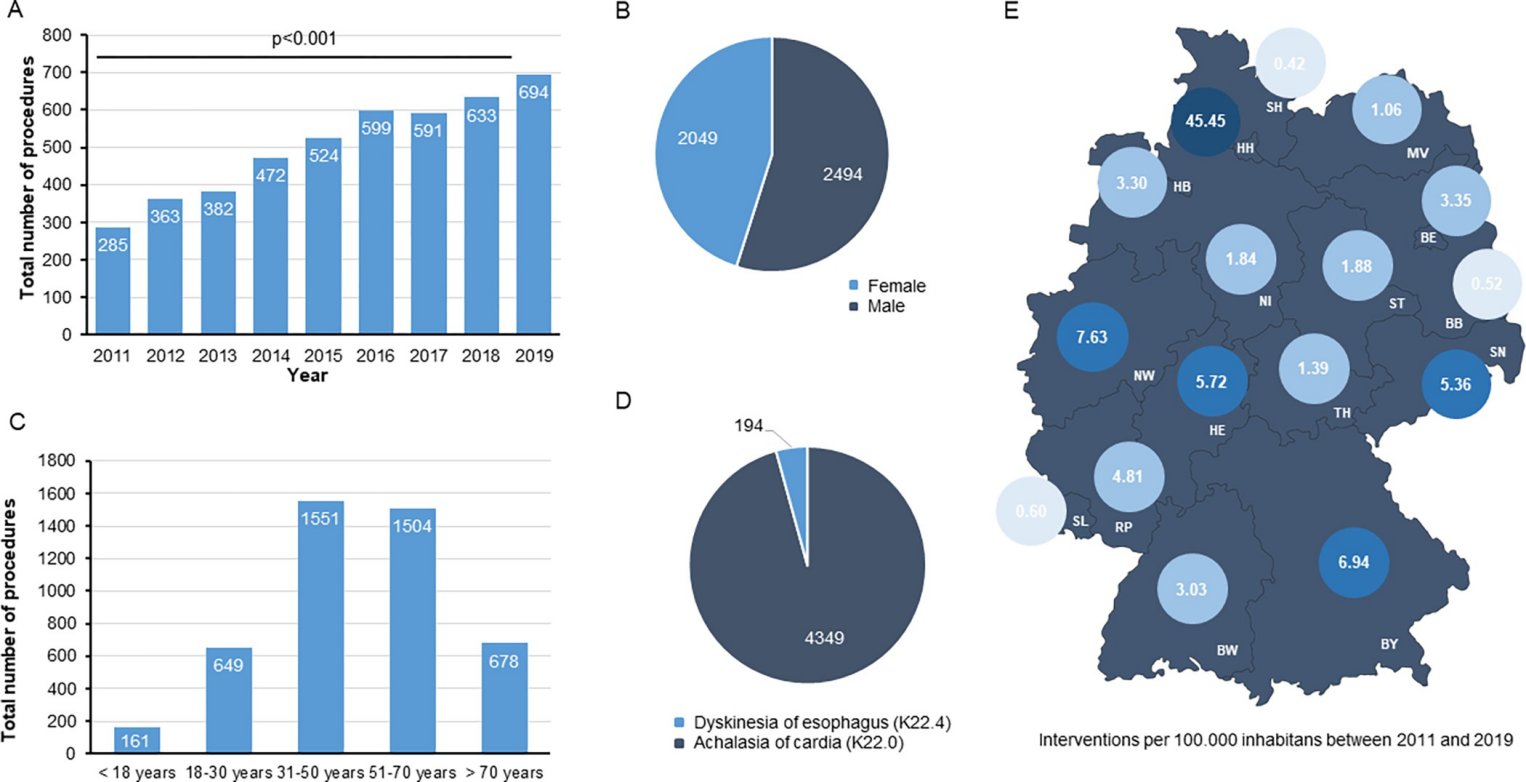
Invasive treatment for esophageal motility disorder in Germany between 2011 and 2019. (A) The total number of performed procedures increases between 2011 and 2019 over time. (B) Most patients are male (54.9%). (C) Most patients are aged between 31 and 70 years. (D) Achalasia of cardia (K22.0) represents the major treatment indication (95.7%). (E) There are significant differences regarding the regional distribution of performed procedures (BB: Brandenburg, BE: Berlin, BW: Baden-Württemberg, BY: Bavaria, HE: Hesse, HB: Bremen, HH: Hamburg, MV: Mecklenburg-Western Pomerania, NI: Lower Saxony, NW: North Rhine-Westphalia, RP: Rhineland-Palatinate, SH: Schleswig-Holstein, SL: Saarland, SN: Saxony, ST: Saxony-Anhalt, TH: Thuringia).

We first aimed at gaining an overview of the invasive treatment landscape for esophageal motility disorders in Germany within the last decade. Interestingly, the total number of performed procedures significantly increased over time and more than doubled from 285 procedures in 2011 to 694 procedures in 2019 (Fig 1A). The majority of patients were male (54.9%, Fig 1B) and aged between 31 and 70 years (Fig 1C). About 15% of patients (n = 678) received invasive treatment for esophageal motility disorders at an age above 70 years (Fig 1C). The mean patients’ age significantly increased over time from 44.7 years in 2011 to 49.6 years in 2019 (S1 Fig). Achalasia of cardia (K22.0) represented the main treatment indication (95.7%, Fig 1D). Notably, we observed significant differences regarding the regional distribution of performed procedures (Fig 1E). As such, the number of procedures per 100.000 inhabitants were highest in Hamburg (45.45), North Rhine-Westphalia (7.63) and Bavaria (6.94), while invasive treatment for esophageal motility disorders was decisively less common in Saarland (0.60), Brandenburg (0.52) or Saxony-Anhalt (0.42). Tables 1 and S1 provide a detailed overview on the study population.

### Surgical vs. endoscopic treatment of esophageal motility disorder

**Fig 2:**
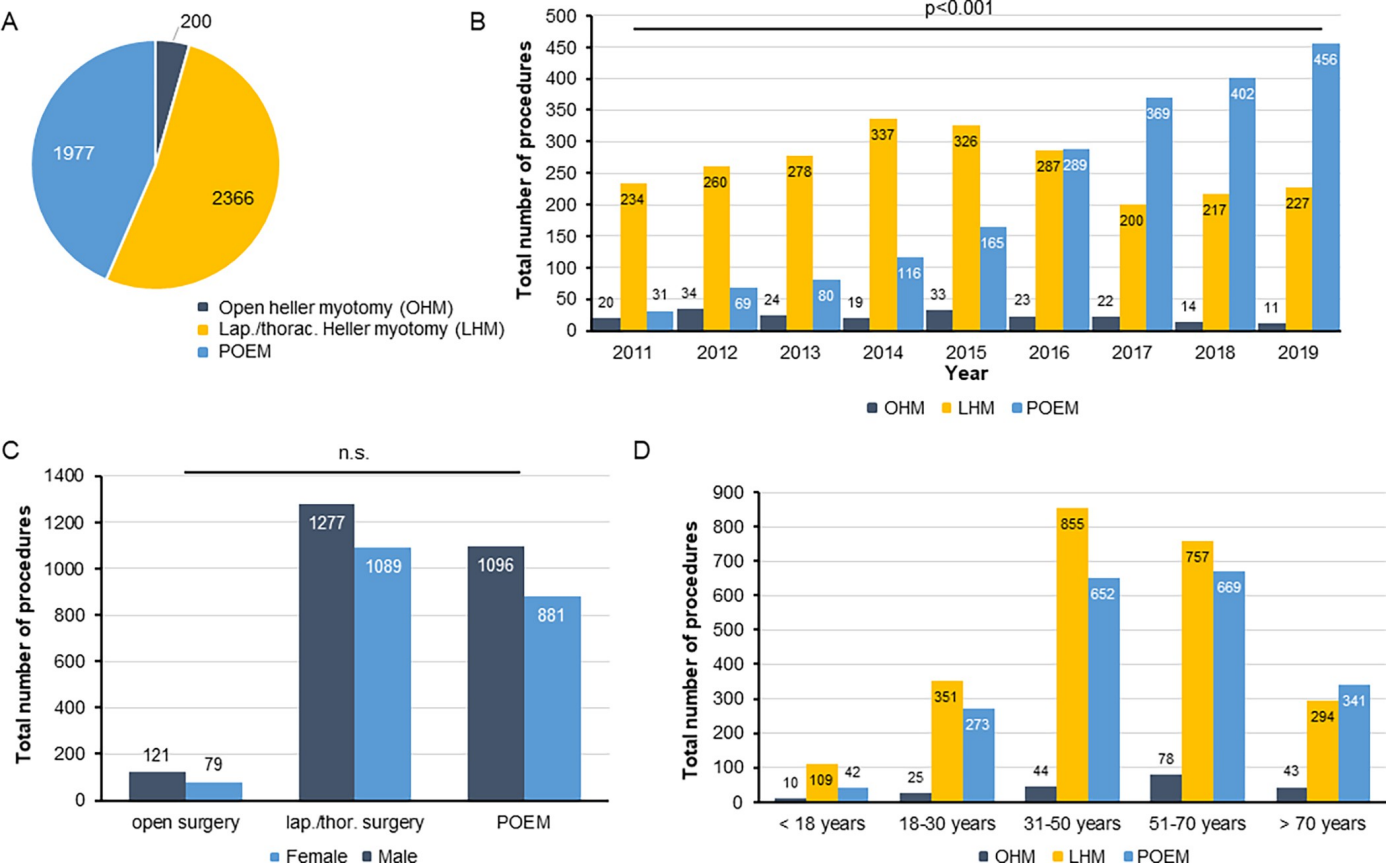
Surgical vs. endoscopic treatment of esophageal motility disorder. (A) Within the study period, the majority of patients (52.1%) underwent LHM followed by POEM (43.5%), while OHM was only rarely performed (4.4%). (B) The relative proportion of POEM shows a stepwise increase from only 10.9% in 2011 to 65.7% in 2019, with 2016 being the year POEM surpassed LHM in number for the first time. (C) The sex ratio of each procedure is comparable. (D) There is a higher proportion of POEM in patients older than 70 years (50.3%), compared to younger age groups (<18 years: 26.1%, 18–30 years: 42.1%, 31–50 years: 42.0%, 51–70 years: 44.4%).

Next, we compared the frequency of the three major invasive treatment strategies for esophageal motility disorders in Germany (open Heller myotomy (OHM), laparoscopic/ thoracoscopic Heller myotomy (LHM) and per-oral endoscopic myotomy (POEM, see Materials & Methods for details). Within the total study period, the majority of patients (52.1%) underwent LHM followed by POEM (43.5%), while OHM was only rarely performed (4.4%, Fig 2A). Interestingly, we observed a significant increase in the number of patients undergoing POEM during the observation period. The relative proportion of POEM showed a stepwise increase from only 10.9% in 2011 to 65.7% in 2019, with 2016 being the year POEM surpassed LHM in number for the first time (Fig 2B). The sex ratio of each procedure was comparable (Fig 2C). When comparing the frequency of the three procedure between different age groups, we observed a higher proportion of POEM in patients older than 70 years (50.3%), compared to younger age groups (<18 years: 26.1%, 18–30 years: 42.1%, 31–50 years: 42.0%, 51–70 years: 44.4%).

### Evaluation of the post-interventional clinical course following invasive treatment for esophageal motility disorder

**Fig 3:**
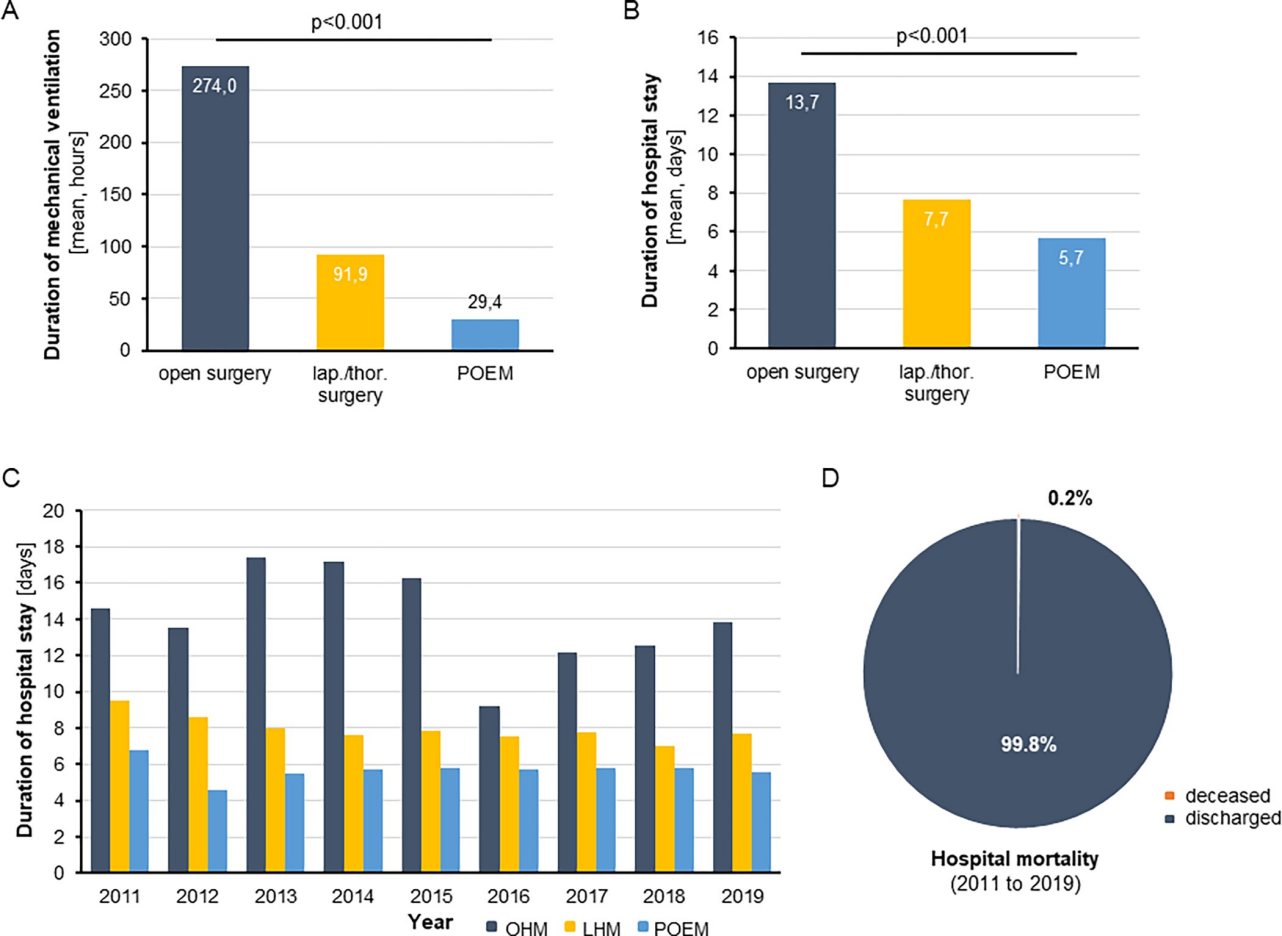
Post-interventional clinical course following invasive treatment for esophageal motility disorder. (A) The mean duration of post-interventional mechanical ventilation is significantly lower in patients undergoing POEM (29.4 hours) compared to OHM (274.0 hours) and LHM (91.9 hours). (B) The mean duration of hospitalization is 5.7 days following POEM, which is significantly lower compared to patients undergoing OHM (13.7 days) or LHM (7.7 days). (C) The duration of hospitalization decreased from 9.5 days (2011) to 7.7 days (2019) for LHM and remained unchanged with respect to OHM and POEM. (D) The overall hospital mortality of all procedure combined was 0.2%.

In order to dissect potential differences in the individual post-interventional course of patients with esophageal motility disorder between the different treatment approaches, we subsequently evaluated the duration of post-interventional mechanical ventilation (MV) and hospitalization. Importantly, we observed a significantly lower mean duration of post-interventional MV in patients undergoing POEM (29.4 hours) compared to both OHM (274.0 hours) and LHM (91.9 hours, Fig 3A). In line, there was a significant difference in terms of the duration of hospital stay between the procedures. As such, the mean duration of hospitalization was only 5.7 days following POEM, which was significantly lower compared to patients undergoing OHM (13.7 days) or LHM (7.7 days, Fig 3B). When looking at the duration of hospitalization over time, we observed a significant reduction from 9.5 days (2011) to 7.7 days (2019) with respect to LHM (p = 0.008), while there was no significant change for OHM or POEM during the observation period. The overall hospital mortality of all procedure combined was only 0.2% (Fig 3D). A detailed comparison of hospital mortality between procedures was not feasible due to small number of events and the strict anonymization measures of the Federal Statistical Office.

## Discussion

PEMDs significantly affect the quality of life of patients, as the leading symptoms of dysphagia, regurgitations, weight loss, and pain may result in psychosocial impairment in addition to the primary disabling features. In a recently published study, we could demonstrate that patients with achalasia suffer from depression significantly more frequently than patients without achalasia, which further limits patient’s quality of life [6]. Consequently, effective and sustainable therapy is essential for affected individuals. Due to their irreversible and in case of achalasia neurodegenerative nature, there are currently no causal therapeutic approaches for PEMDs. All therapeutic measures therefore aim at symptom relief, prevention of disease progression, and avoidance of therapy-associated complications. For achalasia, outstanding improvements in therapeutic approaches have been made in the last decade. With the introduction of POEM in particular, an effective non-surgical endoscopic procedure has become available, which is meanwhile established worldwide as an integral part of the therapeutic spectrum [2,34,35]. Through numerous studies, there is a fairly robust data base for the safety and efficacy of the various therapeutic options. In summary, the two myotomy performing procedures (Heller myotomy and POEM) have the highest efficacy, as demonstrated by two recently published meta-analyses, which compared POEM, LHM, and PD. For this purpose, 6 and 9 randomized controlled trials were analyzed, respectively, comparing the three procedures, which showed equivalence of POEM and LHM and inferiority of PD in treatment response [21,22]. Regarding complications, one meta-analysis showed no difference, whereas in the other, reflux was slightly more common with POEM [21,22]. Despite this apparently obvious data base, recommendations from international professional societies are reluctant to make definitive treatment recommendations [1,2,34,35]. Recommendations are limited to the three modalities PD, LHM and POEM which are mentioned as interventions with comparable efficacies for therapy-naïve patients; BTX is only considered for patients who are not eligible for more invasive definitive therapy due to comorbidities or limited life expectancy. The choice between the three different therapeutic modalities should therefore depend on patient-specific characteristics, patient preference, manometric subtype, potential complications, secondary post-therapeutic conditions (such as reflux disease, Barrett’s esophagus, esophageal cancer), and the experience of the particular institution, and should include a detailed explanation to the patient of the advantages and disadvantages of each procedure.

For the treatment of SEDs, the situation is different. Here, no high-quality studies are available that would justify a clear therapy recommendation based on evidence. However, since there is overlap with achalasia (in particular with type III) in pathophysiology and symptoms, patients with SEDs are usually treated with the same therapeutic modalities as for achalasia. Thus, after the introduction of POEM, it seemed logical to treat SEDs, which were difficult to treat anyway, with this novel effective procedure. Preliminary smaller studies, mostly retrospective or uncontrolled, indicate a high level of efficacy and safety [25–30,32,36–38]. Nevertheless, due to insufficient high-quality data, POEM should currently be considered with caution for the treatment of SEDs, providing there has been careful diagnostic workup, exhaustion of other therapeutic options, including pharmacologic therapy, interdisciplinary team discussion and detailed patient informed consent. Since the guideline recommendations for achalasia consider the two most effective methods (POEM, LHM) as equivalent, first promising data for the use of POEM for the treatment of SEDs are available, and no data on the current reality of PEMDs treatment in Germany are existent so far, our aim was to investigate this in more detail.

To our knowledge, our study is the first comprehensive analysis evaluating invasive treatment options for PEMDs in Germany. By analyzing a total of 4543 hospitalized PEMD cases, which were predominantly (95.7%) achalasia patients, we show that the total number of invasively treated PEMD cases more than doubled between 2011 and 2019. Interestingly, we observed a large geographical heterogeneity with respect to the total number of interventions performed that ranged from 0.60 to 45.45 per 100,000 inhabits. This observation reflects the high level of technical expertise required in the invasive treatment of PEMDs, which leads to a concentration of experts in centers of expertise that are not geographically balanced in Germany. We show that there was a steady increase in POEM cases since its introduction in Germany in 2011, which even became the most commonly performed procedure from 2016. This trend, which is likewise observed in other geographical regions worldwide [39], is most likely ongoing until today. Another important aspect which can be observed is that patients treated by POEM require significantly shorter post-interventional ventilation times and can be discharged from the hospital more quickly than patients treated surgically. In contrast, the overall in-hospital mortality of all procedures was very low (0.2%), which precluded comparative analysis of the different procedures.

Our observation of an increase in overall treatment rates for PEMDs is consistent with the fact that the prevalence of PEMDs, and in particular achalasia with its low mortality, is increasing in the aging population [40]. This can certainly be attributed to the widespread use of HRM, but probably also to growing awareness among physicians to these overall rare diseases. Some authors mention concern that the use of HRM prior to the introduction of the CCv4 in 2020 may have caused overdiagnosis of PEMDs and consequently overtreatment, particularly in the form of POEM [41]. Since the changes in CCv4 mainly refer to type III achalasia and EGJOO and we could not further differentiate the diagnoses "achalasia" and "dyskinesia of the esophagus" in its subtypes in our work, the assumption of a PEMD overdiagnosis cannot be confirmed but also not definitely excluded. However, a relevant bias in diagnosis seems unlikely because type III achalasia is the rarest form of achalasia, accounting for approximately 10–15% of cases [42], and non-achalasia PEMDs, which include EGJOO, accounted for only 4.3% of all cases in our analysis. What might speak for the hypothesis of a PEMD overdiagnosis with the result of increasing POEM rates is the fact that we were able to show that the increase in total intervention numbers was mainly driven by an increase in POEMs, with OHM and LHM rates remaining relatively stable over the study period. On the other hand, the increase in POEMs may also reflect a preference by many patients for the endoscopic, less invasive procedure over surgical alternatives. Additionally, there are hints in our analysis that an increase in the number of interventions could also be explained by an increase in the treatment of older patients. Firstly, a significant increase in the average age of patients was observed during the study period, and secondly, 15% of all patients treated were in the age group >70 years. In this elderly age group, the most frequently performed form of treatment was POEM. This could be explained by the lower invasiveness of POEM compared to the surgical procedures and supports the observation of smaller studies showing that POEM is a safe and effective treatment modality even in this age group [43,44].

Furthermore, we demonstrate that patients treated with POEM required significantly less post-interventional mechanical ventilation and were hospitalized significantly shorter than patients treated surgically. These two aspects may also indicate the lower invasiveness of POEM compared to surgery and are in line with the results of a recently published surgical study, which showed that patients who underwent POEM had better perioperative outcomes in terms of shorter operative room time, less estimated blood loss, and shorter length of stay compared with patients who underwent LHM [45]. Furthermore, POEM patients had less pain at discharge, stopped taking narcotic analgesics earlier, and returned to their activities of daily living earlier than patients who underwent LHM [45]. In addition, there are studies showing that POEM is sufficiently safe to be performed on an outpatient basis in a large number of cases (48–62%) [46,47], which highlights its minimal invasive nature even compared to laparoscopic surgery and may contribute to even more patients opting for this procedure in the future. These aspects regarding patient safety, shorter ventilation, shorter hospital stay or even the possibility of outpatient treatment may certainly contribute to the fact that POEM can be classified as more cost-effective compared to surgical procedures, as several studies have recently demonstrated [45,48–51]. Overall, the mean duration of post-interventional ventilation in the POEM group (29.4 hours) seems quite high to us, since in our experience the majority of patients can be extubated in the intervention room immediately after POEM. Whether these relatively long postinterventional ventilation times are attributable to the first years after the introduction of the procedure, in which ventilation may have been continued postinterventionally as a prophylactic measure for safety reasons, or necessarily had to be continued because of higher intraprocedural complication rates, cannot be answered.

Our study has several limitations inherent to database analysis research. Most importantly, no information on coding quality is available and the database is not subject to systematic quality control between hospitals. Diagnoses are coded using ICD-10 codes, which can lead to misclassification and undercoding of certain diagnoses. For example, there is no information on whether the coding of "dyskinesia of the esophagus" actually includes diseases such as JE, DES, and EGJOO, which can now be classified with HRM and CCv4. Regardless of coding quality, accurate identification of these non-Achalasia PEMDs is very difficult anyway, as definitions have changed steadily over time [7,52]. Furthermore, OHM, LHM, and POEM have been identified using OPS codes. What may have led to a bias in the data in this regard is the fact that, after the introduction of POEM in 2011, there was no specific OPS code for this new type of procedure initially. Therefore, the codes used for POEM were 5–420.06 and 5–420.26, which until then had been used for "simple endoscopic esophageal / esophagogastric myotomy" without submucosal tunneling, meaning full-thickness myotomy. Thus, it cannot be excluded that among the cases we enrolled were not only POEMs but also cases of this "simple full-thickness myotomy". However, since this procedure, which was introduced in the 1970s, is of no relevance in clinical practice nowadays, the bias is likely to be minor. Nevertheless, as the use of codes 5–420.06 and 5–420.26 did not represent the significantly higher expense of POEM compared with "simple full-thickness myotomy" in the German DRG system, it cannot be ruled out that some centers may have used other codes in the first years, such as 5–420.0x or 5–420.2x (“other access”), in order to obtain higher per-case charges for POEM patients. As we believe that the additional evaluation of these codes, which are no longer used for POEM today, would have caused further imprecision in the data, we decided against the inclusion of the codes 5–420.0x and 5–420.2x in our analysis. In addition, although nationwide systematic databases offer the potential for statistically reliable large-scale data analysis, the depth of available parameters is often limited and important clinical factors such as socioeconomic status are missing. Finally, the small number of deaths and cases needing prolonged mechanical ventilation in the total cohort prevented a dedicated subgroup analysis of these parameters between the individual treatment methods, as the legal restrictions of the Federal Statistical Office had to be respected with regard to a potential threat to anonymity.

In conclusion, our data provide a systematic overview of the invasive treatment landscape for PEMDs in Germany over the last decade. We demonstrate the increasing importance of POEM as a minimally invasive endoscopic procedure, appearing to emerge as the predominantly performed treatment. POEM is associated with shorter post-interventional ventilation times and hospital length of stay, supporting the already reported data regarding safety, efficacy, and cost-effectiveness. The combined in-hospital mortality of all procedures is very low, highlighting the high safety profile of all invasive procedures for the treatment of PEMDs.

## Supporting information

S1 Fig Development of patients’ age during the study period. (A) The mean patients’ age increases over time from 44.7 years in 2011 to 49.6 years in 2019.
(TIF)

S1 Table Detailed description of study population from 2011 to 2019. (DOCX)

## References

[1] Weusten B, Barret M, Bredenoord AJ, Familiari P, Gonzalez JM, van Hooft JE, et al. Endoscopic management of gastrointestinal motility disorders—part 1: European Society of Gastrointestinal Endoscopy (ESGE) Guideline. Endoscopy. 2020;52(6):498–515. Epub 2020/05/07. doi: 10.1055/a-1160-5549 . 32375192

[2] Oude Nijhuis RAB, Zaninotto G, Roman S, Boeckxstaens GE, Fockens P, Langendam MW, et al. European guidelines on achalasia: United European Gastroenterology and European Society of Neurogastroenterology and Motility recommendations. United European Gastroenterol J. 2020;8(1):13–33. Epub 2020/03/28. doi: 10.1177/2050640620903213 ; PubMed Central PMCID: PMC7005998. 32213062 PMC7005998

[3] Boeckxstaens GE, Zaninotto G, Richter JE. Achalasia. Lancet. 2014;383(9911):83–93. Epub 2013/07/23. doi: 10.1016/S0140-6736(13)60651-0 . 23871090

[4] Gockel I, Becker J, Wouters MM, Niebisch S, Gockel HR, Hess T, et al. Common variants in the HLA-DQ region confer susceptibility to idiopathic achalasia. Nat Genet. 2014;46(8):901–4. Epub 2014/07/07. doi: 10.1038/ng.3029 . 24997987

[5] Jeon HH, Kim JH, Youn YH, Park H, Conklin JL. Clinical Characteristics of Patients with Untreated Achalasia. J Neurogastroenterol Motil. 2017;23(3):378–84. Epub 2017/03/30. doi: 10.5056/jnm16177 ; PubMed Central PMCID: PMC5503287. 28351117 PMC5503287

[6] Loosen SH, Kandler J, Luedde T, Kostev K, Roderburg C. Achalasia is associated with a higher incidence of depression in outpatients in Germany. PLoS One. 2021;16(4):e0250503. Epub 2021/05/01. doi: 10.1371/journal.pone.0250503 ; PubMed Central PMCID: PMC8087033 but IQVIA did not fund this research. The specific roles of this author are articulated in the ’author contributions’ section. PMC8087033 33930060

[7] Yadlapati R, Kahrilas PJ, Fox MR, Bredenoord AJ, Prakash Gyawali C, Roman S, et al. Esophageal motility disorders on high-resolution manometry: Chicago classification version 4.0((c)). Neurogastroenterol Motil. 2021;33(1):e14058. Epub 2020/12/30. doi: 10.1111/nmo.14058 ; PubMed Central PMCID: PMC8034247. 33373111 PMC8034247

[8] Philonenko S, Roman S, Zerbib F, Gourcerol G, Gault N, Ropert A, et al. Jackhammer esophagus: Clinical presentation, manometric diagnosis, and therapeutic results-Results from a multicenter French cohort. Neurogastroenterol Motil. 2020;32(11):e13918. Epub 2020/06/09. doi: 10.1111/nmo.13918 . 32510747

[9] Herregods TV, Smout AJ, Ooi JL, Sifrim D, Bredenoord AJ. Jackhammer esophagus: Observations on a European cohort. Neurogastroenterol Motil. 2017;29(4). Epub 2016/10/19. doi: 10.1111/nmo.12975 . 27753176

[10] Ponds FA, Fockens P, Lei A, Neuhaus H, Beyna T, Kandler J, et al. Effect of Peroral Endoscopic Myotomy vs Pneumatic Dilation on Symptom Severity and Treatment Outcomes Among Treatment-Naive Patients With Achalasia: A Randomized Clinical Trial. JAMA. 2019;322(2):134–44. Epub 2019/07/10. doi: 10.1001/jama.2019.8859 . 31287522 PMC6618792

[11] Boeckxstaens GE, Annese V, des Varannes SB, Chaussade S, Costantini M, Cuttitta A, et al. Pneumatic dilation versus laparoscopic Heller’s myotomy for idiopathic achalasia. N Engl J Med. 2011;364(19):1807–16. Epub 2011/05/13. doi: 10.1056/NEJMoa1010502 . 21561346

[12] Allaix ME, Patti MG. Heller myotomy for achalasia. From the open to the laparoscopic approach. World J Surg. 2015;39(7):1603–7. doi: 10.1007/s00268-014-2914-3 . 25526923

[13] Werner YB, Hakanson B, Martinek J, Repici A, von Rahden BHA, Bredenoord AJ, et al. Endoscopic or Surgical Myotomy in Patients with Idiopathic Achalasia. N Engl J Med. 2019;381(23):2219–29. Epub 2019/12/05. doi: 10.1056/NEJMoa1905380 . 31800987

[14] Moonen A, Annese V, Belmans A, Bredenoord AJ, Bruley des Varannes S, Costantini M, et al. Long-term results of the European achalasia trial: a multicentre randomised controlled trial comparing pneumatic dilation versus laparoscopic Heller myotomy. Gut. 2016;65(5):732–9. Epub 20151127. doi: 10.1136/gutjnl-2015-310602 . 26614104

[15] Inoue H, Minami H, Kobayashi Y, Sato Y, Kaga M, Suzuki M, et al. Peroral endoscopic myotomy (POEM) for esophageal achalasia. Endoscopy. 2010;42(4):265–71. Epub 20100330. doi: 10.1055/s-0029-1244080 . 20354937

[16] Vespa E, Pellegatta G, Thoguluva Chandrasekar V, Spadaccini M, Patel HK, Maselli R, et al. Long-Term Outcomes of Per-Oral Endoscopic Myotomy (POEM) for Achalasia: a Systematic Review and Meta-analysis. Endoscopy. 2022. Epub 20220707. doi: 10.1055/a-1894-0147 . 35798336

[17] Modayil RJ, Zhang X, Rothberg B, Kollarus M, Galibov I, Peller H, et al. Peroral endoscopic myotomy: 10-year outcomes from a large, single-center U.S. series with high follow-up completion and comprehensive analysis of long-term efficacy, safety, objective GERD, and endoscopic functional luminal assessment. Gastrointest Endosc. 2021;94(5):930–42. Epub 2021/05/15. doi: 10.1016/j.gie.2021.05.014 . 33989646

[18] Campagna RAJ, Cirera A, Holmstrom AL, Triggs JR, Teitelbaum EN, Carlson DA, et al. Outcomes of 100 Patients More Than 4 Years After POEM for Achalasia. Ann Surg. 2021;273(6):1135–40. Epub 2021/04/30. doi: 10.1097/SLA.0000000000004830 ; PubMed Central PMCID: PMC8260096. 33914488 PMC8260096

[19] Kuipers T, Ponds FA, Fockens P, Bastiaansen BAJ, Lei A, Oude Nijhuis RAB, et al. Peroral endoscopic myotomy versus pneumatic dilation in treatment-naive patients with achalasia: 5-year follow-up of a randomised controlled trial. Lancet Gastroenterol Hepatol. 2022;7(12):1103–11. Epub 20221004. doi: 10.1016/S2468-1253(22)00300-4 . 36206786

[20] Zhang XC, Li QL, Xu MD, Chen SY, Zhong YS, Zhang YQ, et al. Major perioperative adverse events of peroral endoscopic myotomy: a systematic 5-year analysis. Endoscopy. 2016;48(11):967–78. Epub 20160722. doi: 10.1055/s-0042-110397 . 27448052

[21] Mundre P, Black CJ, Mohammed N, Ford AC. Efficacy of surgical or endoscopic treatment of idiopathic achalasia: a systematic review and network meta-analysis. Lancet Gastroenterol Hepatol. 2021;6(1):30–8. Epub 2020/10/10. doi: 10.1016/S2468-1253(20)30296-X . 33035470

[22] Facciorusso A, Singh S, Abbas Fehmi SM, Annese V, Lipham J, Yadlapati R. Comparative efficacy of first-line therapeutic interventions for achalasia: a systematic review and network meta-analysis. Surg Endosc. 2021;35(8):4305–14. Epub 20200827. doi: 10.1007/s00464-020-07920-x ; PubMed Central PMCID: PMC8011535. 32856150 PMC8011535

[23] Zerbib F, Roman S. Current Therapeutic Options for Esophageal Motor Disorders as Defined by the Chicago Classification. J Clin Gastroenterol. 2015;49(6):451–60. doi: 10.1097/MCG.0000000000000317 . 25844840

[24] Leconte M, Douard R, Gaudric M, Dumontier I, Chaussade S, Dousset B. Functional results after extended myotomy for diffuse oesophageal spasm. Br J Surg. 2007;94(9):1113–8. doi: 10.1002/bjs.5761 . 17497756

[25] Canakis A, Xie G, Kim RE. Peroral Endoscopic Myotomy Is an Effective Treatment Option for Managing Jackhammer Esophagus: A Single Center Experience. J Clin Gastroenterol. 2022. Epub 20220428. doi: 10.1097/MCG.0000000000001717 . 35537134

[26] Nabi Z, Chavan R, Ramchandani M, Basha J, Jagtap N, Karyampudi A, et al. Long-term Outcomes of Per-oral Endoscopic Myotomy in Spastic Esophageal Motility Disorders: A Large, Single-Center Study. J Clin Gastroenterol. 2021;55(7):594–601. doi: 10.1097/MCG.0000000000001395 . 32657960

[27] Filicori F, Dunst CM, Sharata A, Abdelmoaty WF, Zihni AM, Reavis KM, et al. Long-term outcomes following POEM for non-achalasia motility disorders of the esophagus. Surg Endosc. 2019;33(5):1632–9. Epub 20180919. doi: 10.1007/s00464-018-6438-z . 30232618

[28] Khashab MA, Familiari P, Draganov PV, Aridi HD, Cho JY, Ujiki M, et al. Peroral endoscopic myotomy is effective and safe in non-achalasia esophageal motility disorders: an international multicenter study. Endoscopy international open. 2018;6(8):E1031–E6. Epub 2018/08/15. doi: 10.1055/a-0625-6288 ; PubMed Central PMCID: PMC6086680. 30105290 PMC6086680

[29] Albers D, Frieling T, Dakkak D, Kuhlbusch-Zicklam R, Tox U, Gittinger M, et al. Peroral endoscopic myotomy (POEM) is effective in treatment of noncardiac chest pain caused by hypercontractile esophageal motility disorders: results of the POEM-HYPE-Study. Z Gastroenterol. 2018;56(11):1337–42. Epub 2018/10/09. doi: 10.1055/a-0668-2605 . 30296811

[30] Khashab MA, Messallam AA, Onimaru M, Teitelbaum EN, Ujiki MB, Gitelis ME, et al. International multicenter experience with peroral endoscopic myotomy for the treatment of spastic esophageal disorders refractory to medical therapy (with video). Gastrointest Endosc. 2015;81(5):1170–7. Epub 20150126. doi: 10.1016/j.gie.2014.10.011 . 25634487

[31] Stavropoulos SN, Modayil RJ, Friedel D, Savides T. The International Per Oral Endoscopic Myotomy Survey (IPOEMS): a snapshot of the global POEM experience. Surg Endosc. 2013;27(9):3322–38. Epub 20130403. doi: 10.1007/s00464-013-2913-8 . 23549760

[32] Ichkhanian Y, Sanaei O, Canakis A, Vosoughi K, Almazan E, Ghandour B, et al. Esophageal peroral endoscopic myotomy (POEM) for treatment of esophagogastric junction outflow obstruction: results from the first prospective trial. Endoscopy international open. 2020;8(9):E1137–E43. Epub 20200831. doi: 10.1055/a-1198-4643 ; PubMed Central PMCID: PMC7458721. 32904698 PMC7458721

[33] Lee SW. Methods for testing statistical differences between groups in medical research: statistical standard and guideline of Life Cycle Committee. Life Cycle. 2022;2:e1. doi: 10.54724/lc.2022.e1

[34] Kohn GP, Dirks RC, Ansari MT, Clay J, Dunst CM, Lundell L, et al. SAGES guidelines for the use of peroral endoscopic myotomy (POEM) for the treatment of achalasia. Surg Endosc. 2021;35(5):1931–48. Epub 20210209. doi: 10.1007/s00464-020-08282-0 . 33564964

[35] Vaezi MF, Pandolfino JE, Yadlapati RH, Greer KB, Kavitt RT. ACG Clinical Guidelines: Diagnosis and Management of Achalasia. Am J Gastroenterol. 2020;115(9):1393–411. doi: 10.14309/ajg.0000000000000731 . 32773454 PMC9896940

[36] Masadeh M, Nau P, Chandra S, Klair J, Keech J, Parekh K, et al. Experience with Peroral Endoscopic Myotomy for Achalasia and Spastic Esophageal Motility Disorders at a Tertiary U.S. Center. Clin Endosc. 2020;53(3):321–7. Epub 20191120. doi: 10.5946/ce.2019.110 ; PubMed Central PMCID: PMC7280846. 31744270 PMC7280846

[37] Bernardot L, Roman S, Barret M, Vitton V, Wallenhorst T, Pioche M, et al. Efficacy of per-oral endoscopic myotomy for the treatment of non-achalasia esophageal motor disorders. Surg Endosc. 2020;34(12):5508–15. Epub 20200113. doi: 10.1007/s00464-019-07348-y . 31932930

[38] Bechara R, Ikeda H, Inoue H. Peroral endoscopic myotomy for Jackhammer esophagus: to cut or not to cut the lower esophageal sphincter. Endoscopy international open. 2016;4(5):E585–8. Epub 20160408. doi: 10.1055/s-0042-105204 ; PubMed Central PMCID: PMC4892003. 27274539 PMC4892003

[39] Trieu JA, Dua A, Enofe I, Shastri N, Venu M. Population trends in achalasia diagnosis and management: a changing paradigm. Dis Esophagus. 2021;34(5). doi: 10.1093/dote/doab014 . 33728431

[40] Samo S, Carlson DA, Gregory DL, Gawel SH, Pandolfino JE, Kahrilas PJ. Incidence and Prevalence of Achalasia in Central Chicago, 2004–2014, Since the Widespread Use of High-Resolution Manometry. Clin Gastroenterol Hepatol. 2017;15(3):366–73. Epub 2016/09/02. doi: 10.1016/j.cgh.2016.08.030 ; PubMed Central PMCID: PMC5316341. 27581064 PMC5316341

[41] Herbella FAM, Del Grande LM, Schlottmann F, Patti MG. Changes in the Treatment of Primary Esophageal Motility Disorders Imposed by the New Classification for Esophageal Motility Disorders on High Resolution Manometry (Chicago Classification 4.0). Adv Ther. 2021;38(5):2017–26. Epub 20210327. doi: 10.1007/s12325-021-01714-w ; PubMed Central PMCID: PMC8107150. 33772739 PMC8107150

[42] Rohof WO, Salvador R, Annese V, Bruley des Varannes S, Chaussade S, Costantini M, et al. Outcomes of treatment for achalasia depend on manometric subtype. Gastroenterology. 2013;144(4):718–25; quiz e13-4. Epub 20121228. doi: 10.1053/j.gastro.2012.12.027 . 23277105

[43] Nakamura J, Hikichi T, Hashimoto M, Takasumi M, Kato T, Kobashi R, et al. Efficacy and Safety of Peroral Endoscopic Myotomy for Esophageal Achalasia and Achalasia-Related Diseases in Patients Aged 75 Years and Over. Healthcare (Basel). 2021;9(12). Epub 2021/12/25. doi: 10.3390/healthcare9121668 ; PubMed Central PMCID: PMC8700855. 34946392 PMC8700855

[44] Chen YI, Inoue H, Ujiki M, Draganov PV, Colavita P, Mion F, et al. An international multicenter study evaluating the clinical efficacy and safety of per-oral endoscopic myotomy in octogenarians. Gastrointest Endosc. 2018;87(4):956–61. Epub 20170221. doi: 10.1016/j.gie.2017.02.007 . 28235595

[45] Attaar M, Su B, Wong HJ, Kuchta K, Denham W, Linn JG, et al. Comparing cost and outcomes between peroral endoscopic myotomy and laparoscopic heller myotomy. Am J Surg. 2021;222(1):208–13. Epub 2020/11/10. doi: 10.1016/j.amjsurg.2020.10.037 . 33162014

[46] Attaar M, Su B, Wong HJ, Kuchta K, Denham W, Haggerty SP, et al. Factors associated with admission after implementation of a same-day discharge pathway in patients undergoing peroral endoscopic myotomy (POEM). Surg Endosc. 2021;35(7):3971–80. Epub 20200811. doi: 10.1007/s00464-020-07866-0 . 32780244

[47] Benias PC, Korrapati P, Raphael KL, D’Souza LS, Inamdar S, Trindade AJ, et al. Safety and feasibility of performing peroral endoscopic myotomy as an outpatient procedure with same-day discharge. Gastrointest Endosc. 2019;90(4):570–8. Epub 20190510. doi: 10.1016/j.gie.2019.04.247 . 31078571

[48] Conte TM, Haddad LBP, Ribeiro IB, de Moura ETH, DʼAlbuquerque LAC, de Moura EGH. Peroral endoscopic myotomy (POEM) is more cost-effective than laparoscopic Heller myotomy in the short term for achalasia: economic evaluation from a randomized controlled trial. Endoscopy international open. 2020;8(11):E1673–e80. Epub 2020/11/04. doi: 10.1055/a-1261-3417 ; PubMed Central PMCID: PMC7584466. 33140023 PMC7584466

[49] Wirsching A, Boshier PR, Klevebro F, Kaplan SJ, Seesing MF, El-Moslimany R, et al. Comparison of costs and short-term clinical outcomes of per-oral endoscopic myotomy and laparoscopic Heller myotomy. Am J Surg. 2019;218(4):706–11. Epub 20190718. doi: 10.1016/j.amjsurg.2019.07.026 . 31353034

[50] Shah ED, Chang AC, Law R. Valuing innovative endoscopic techniques: per-oral endoscopic myotomy for the management of achalasia. Gastrointest Endosc. 2019;89(2):264–73 e3. Epub 20180421. doi: 10.1016/j.gie.2018.04.2341 . 29684386

[51] Greenleaf EK, Winder JS, Hollenbeak CS, Haluck RS, Mathew A, Pauli EM. Cost-effectiveness of per oral endoscopic myotomy relative to laparoscopic Heller myotomy for the treatment of achalasia. Surg Endosc. 2018;32(1):39–45. Epub 20171207. doi: 10.1007/s00464-017-5629-3 . 29218664

[52] Kahrilas PJ, Bredenoord AJ, Fox M, Gyawali CP, Roman S, Smout AJ, et al. The Chicago Classification of esophageal motility disorders, v3.0. Neurogastroenterol Motil. 2015;27(2):160–74. Epub 2014/12/04. doi: 10.1111/nmo.12477 ; PubMed Central PMCID: PMC4308501. 25469569 PMC4308501

