# Supplementary figures and images for: Surgical and per-oral endoscopic myotomy (POEM) for the treatment of primary esophageal motility disorders: A systematic analysis of current trends in Germany between 2011 and 2019
Source: PLoS One. 2024 Jan 23;19(1):e0297265. doi: 10.1371/journal.pone.0297265 (PMC10805300; doi:10.1371/journal.pone.0297265)

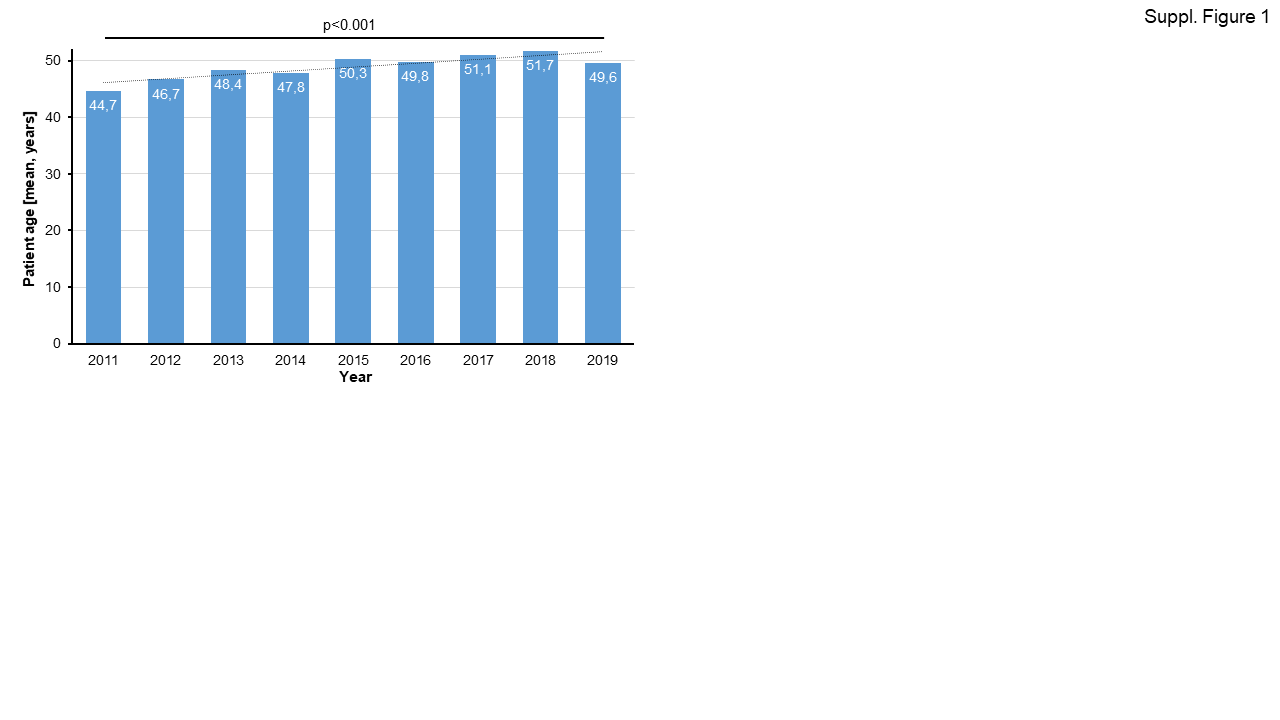

Supplement: S1 Fig — (A) The mean patients’ age increases over time from 44.7 years in 2011 to 49.6 years in 2019. (TIF) [file pone.0297265.s001.TIF]
