# Supplementary material for: Surgical and per-oral endoscopic myotomy (POEM) for the treatment of primary esophageal motility disorders: A systematic analysis of current trends in Germany between 2011 and 2019
Source: PLoS One. 2024 Jan 23;19(1):e0297265. doi: 10.1371/journal.pone.0297265 (PMC10805300; doi:10.1371/journal.pone.0297265)
Supplement: S1 Table — (DOCX) [file pone.0297265.s002.docx]

# S1 Table. Detailed description of study population from 2011 to 2019

|  | **2011** | **2012** | **2013** | **2014** | **2015** | **2016** | **2017** | **2018** | **2019** |
| --- | --- | --- | --- | --- | --- | --- | --- | --- | --- |
| Number of procedures | | | | | | | | | |
| Total | 285 | 285 | 363 | 382 | 472 | 524 | 599 | 591 | 633 |
| Open heller myotomy (OHM) | 20 | 34 | 24 | 19 | 33 | 23 | 22 | 14 | 11 |
| Lap./thorac. Heller myotomy (LHM) | 234 | 260 | 278 | 337 | 326 | 287 | 200 | 217 | 227 |
| Per-oral endoscopic myotomy (POEM) | 31 | 69 | 80 | 116 | 165 | 289 | 369 | 402 | 456 |
| Sex | | | | | | | | | |
| Male | 149 | 208 | 220 | 258 | 289 | 336 | 299 | 357 | 378 |
| Female | 136 | 155 | 162 | 214 | 235 | 263 | 292 | 276 | 316 |
| Age [years, mean] | 44.7 | 46.7 | 48.4 | 47.8 | 50.3 | 49.8 | 51.1 | 51.7 | 49.6 |
| Number of ventilated patients after procedure | 11 | 7 | 13 | 6 | 10 | 17 | 20 | 17 | 30 |
